# Modified autologous fascial sling technique (‘sling on a string’) for stress incontinence

**DOI:** 10.1007/s00192-021-04815-w

**Published:** 2021-07-14

**Authors:** Victoria Asfour, Kostis I. Nikolopoulos, Giuseppe Alessandro Digesu, Simon Emery, Zainab Khan

**Affiliations:** 1 London North West University Healthcare Trust, Northwick Park Hospital, London, UK; 2 Imperial College London, London, UK; 3 Wexham Park Hospital NHS Foundation Trust, Berkshire, UK; 4 St Mary’s hospital, Imperial College Healthcare Trust, London, UK; 5 Swansea NHS Trust, Swansea, UK; 6 Epsom and St Helier University Hospitals NHS Trust, Epsom, UK

**Keywords:** Sling, Stress incontinence, Autologous sling, Modified Aldridge sling, Sling on a string

## Abstract

**Introduction and hypothesis:**

Describe the modified autologous fascial sling procedure that has been employed in the largest randomized controlled trial comparing autologous slings, mesh slings and xenografts.

**Methods:**

The video aims to demonstrate the modified Aldridge technique. The surgical procedure is demonstrated. A 6-cm suprapubic incision is made to harvest the rectus sheath fascia. Loop-0-PDS sutures are attached on either end of the sling. A marking suture is placed in the middle of the graft to facilitate tension-free adjustment. A vaginal incision is made at the mid-urethra. Paraurethral dissection is performed to create a tunnel for the fascial graft to be passed through (in the same manner as with transvaginal mesh slings). The ends of the graft PDS sutures are passed through the paraurethral tunnel. One hand is placed abdominally below the rectus muscles to palpate the pelvic floor from above. The graft sutures are passed through the pelvic floor with control on either side. A cystoscopy is performed to check the bladder integrity. The graft placement is adjusted to be tenson-free. The incisions are closed. The short- and long-term outcomes of this technique have been investigated and published.

**Results:**

The cure rates and complication rates were no different in the mesh and autologous slings. The xenograft had inferior outcomes.

**Conclusion:**

Autologous fascial slings can be used in the surgical management of urodynamic stress incontinence. The technique demonstrated in this video is the technique employed in the largest randomized controlled trial investigating the efficacy of autologous fascial slings to xenografts and tapes.

**Electronic supplementary material:**

The online version of this article (10.1007/s00192-021-04815-w) contains supplementary material. This video is also available to watch on http://link.springer.com/. Please search for this article by the article title or DOI number, and on the article page click on ‘Supplementary Material’.

## Introduction

Stress urinary incontinence occurs in a fifth of adult women, having a significant impact on the quality of life in about half [1]. A wide range of surgical procedures have been described for the treatment of stress urinary incontinence. Synthetic mid-urethral slings have become the ‘gold standard’ treatment since their introduction in 1995 by Ulmsten and Petros [2]. Currently, the use of synthetic slings for the treatment of stress urinary incontinence is under intense scrutiny. Despite the evidence to support the high success rates and the safety of the synthetic slings, the general public and some health care bodies are expressing concerns regarding the long-term complications and seeking alternative surgical options. In 1933, Philip and Prince first described the autologous sling procedure [3]. Aldridge described an autologous sling using a long piece of fascia that extended from the mid-urethra to the rectus sheath bilaterally [4]. The technique described here was developed by Emery and Lucas in Swansea, UK, in 1990 and was employed in a large randomized controlled trial and other studies [5–8]. It is based on the Aldridge technique. The ‘sling on a string’ uses a small detached piece of fascia, which requires less dissection and is less traumatic [5]. The technique shown in this video uses a bottom-to-top approach, with the sutures tied on either side to the rectus sheath separately instead of in the middle. The sling is adjusted with control and secured tension-free. This is the technique employed in the large randomized controlled trial comparing autologous, xenograft and synthetic slings [6–8]. This technique has been shown to have equivalent outcomes to a synthetic transvaginal tape at over 10 years of follow-up [6]. In 2017, Fusco et al. published a meta-analysis of 15,855 patients having synthetic and autologous fascial slings. They reported similar objective cure rates for both mesh and autologous slings, which were superior to a Burch colposuspension [9].

In the UK, all stress incontinence surgery operations are high-vigilance procedures [10]. Data from these operations should be entered on a national audit database. In the UK, these procedures should be recorded on the British Society of Urogynaecology database (BSUG). Urodynamics should be performed to confirm the diagnosis. The National Institute of Clinical Excellence (NICE) recommends to discuss all the available operations with the patient with the aid of the Patient Decision Tool in the clinic [10]. If the patient chooses a surgical procedure that is not offered locally, then the patient should have the option of being referred to another unit. The patient needs to be fully informed of the short- and long-term outcomes of the procedure, understand what is involved and have enough time to weigh this information and ask questions. Informed consent is a particularly important step of high-vigilance surgery.

## Aim

The video aims to demonstrate step by step a modified surgical technique of an autologous fascial sling in a female patient with stress urinary incontinence.

## Surgical technique

The procedure is undertaken under general anesthesia in the dorsal lithotomy position. A Foley urethral catheter is inserted. A single dose of intravenous antibiotics is administered.

### Abdominal approach

A 6-cm transverse skin incision is made 2 cm above the pubis. A sterilized disposable measuring tape, marker and hand-held monopolar diathermy were used. The fascia is harvested with two parallel transverse incisions approximately 1 cm wide and 8 cm long. The harvested fascia is cleared of the overlying fat. A 0-PDS (polydioxanone) double suture is passed twice through each end of the fascial strip. The suture is cut at a length of approximately 30 cm. A Vicryl 3–0 marking suture is used to mark the middle of the fascial sling. Then, the fascial sling with the sutures is submerged in normal saline to keep it moist during the vaginal dissection in the next step.

### Vaginal approach

Following paraurethral infiltration of local anesthetic (10 ml), xylocaine 1% with adrenaline 100 µg/20 ml (1 in 200,000), a 3-cm midline incision is made through the vaginal mucosa on the anterior vaginal wall, starting at approximately 1 cm proximal to the urethral meatus. The bladder neck is identified by pulling on the Foley catheter and palpating the balloon. The edges of the incised mucosa are grasped with Allis clamps, and caudal traction is applied with support of the index finger of the non-dominant hand to facilitate the dissection of the vaginal mucosa off the underlying pubocervical fascia with Metzenbaum scissors. A tunnel for the insertion of the fascial sling is developed by retracting the lateral edge of the incision with the Allis clamp and keeping the tips of the Metzenbaum scissors pointed toward the patient’s ipsilateral shoulder. The pelvic fascia is not pierced at this stage.

### Insertion of the tape

Once the tape is harvested and the tunnels are created, the surgeon changes gloves. The index and middle fingers of the non-dominant hand are placed at the posterior edge of the pubis through the abdominal incision. Gentle blunt digital dissection of the Retzius space is done to reach the pelvic floor in the midline behind the symphysis pubis. The free ends of the PDS suture on the fascial sling are passed with the tip of a slim long-curved Robert’s clamp. Traction on the catheter helps localize the bladder neck region. The clamp is positioned by the dominant hand vaginally through the paraurethral tunnel until it becomes palpable by the opposite hand’s fingers in the cave of Retzius above the pelvic fascia. The dominant hand then drives the curved Robert’s clamp through to perforate the pelvic fascia from below with digital support from above to control accurate placement and protect the urethra and bladder. The PDS sutures are pulled through the abdominal incision and the process repeated with the fascia and threads contralaterally to create the suburethral support.

At this stage a cystoscopy is performed to assess the integrity of the bladder. Once bladder trauma has been excluded, the PDS sutures are mounted onto a Mayo needle and passed through the ipsilateral rectus sheath 1 cm below the incision and 2 cm lateral to the midline; one thread is then removed and a second bite taken with the remaining single thread and left for tensioning after the sheath is closed. The sling is adjusted vaginally by applying gentle traction on the mid-sling marking suture while both PDS knots are tied. Care at this point is essential to avoid overtightening the PDS and noting that autologous fascia retracts slightly more than mesh tape in the postoperative phase. Once the sling is adjusted and secured, the marking stitch is removed.

Variations of thread carriage and suture tying have not been investigated by a randomized controlled trial. For the technique demonstrated, it is important to ensure symmetrical placement of the graft so that at least 2 cm on each side passes through the endopelvic fascia without tension. Finally, the abdominal and vaginal incisions are closed.

### Postoperatively

The Foley catheter is removed in 4–48 h following the procedure, according to the local protocol.

### Comparison of this technique for autologous fascial sling (AFS) with retropubic mid-urethral sling mesh techniques (MUS-mesh), such as TVT

These techniques are based on the same principles; they have the same mechanism of action and a similar intraoperative complication profile.

The AFS procedure is a longer procedure because the graft needs to be harvested from the abdominal wall instead of using mesh. The vaginal incision and dissection are the same. In MUS-mesh, the mesh is passed mounted on needles retropubically. In MUS-mesh, the urethra is deviated to protect the bladder from injury. In AFS, there is no need to deviate the urethra and bladder base because the sutures are passed with control. Bladder perforation occurs in 4/72 (5.5%) with MUS-mesh and 2/79 (2.5%) with AFS (Kruskall-Wallis, *p* = 0.6) [7]. The urethra and bladder base are protected by the surgeon’s fingers in the cave of Retzius in this demonstrated AFS technique. The sling is adjusted to be tension-free in the same way for both techniques. In MUS-mesh, the mesh holds itself in position after removal of the plastic sheaths. In AFS, the sling sutures need to be secured onto the rectus sheath with less tension than that used for mesh tape. The purpose is to prevent downward displacement not elevation. Correct tension-free adjustment is important for maintaining normal voiding.

Postoperative voiding dysfunction requiring intermittent self-catheterization at 6 weeks postoperatively occurs in 1/67 (1.5%) with TVT and 7/71 (9.9%) with AFS (Kruskall-Wallis, *p* = 0.01) [7]. At 6 months, there is no difference in the voiding function between the two groups: TVT 0/71 and AFS 1/73 (1.4%) (Kruskall-Wallis, *p* = 0.4) [7]. In the long-term (10 years' follow-up), the outcomes of AFS are sustained [6]. The MUS-mesh has the additional risk of mesh complications in the long term.

## Conclusion

The autologous fascial sling procedure is a treatment option for both primary and recurrent stress incontinence in women with high cure rates. With the recent decline in the use of synthetic mesh slings, the autologous fascial sling presents a comparable alternative option.

## Supplementary Information

ESM 1 (MP4 96,608 kb)
